# In-depth analysis of subclass-specific conformational preferences of IgG antibodies

**DOI:** 10.1107/S205225251402209X

**Published:** 2015-01-01

**Authors:** Xinsheng Tian, Bente Vestergaard, Matthias Thorolfsson, Zhiru Yang, Hanne B. Rasmussen, Annette E. Langkilde

**Affiliations:** a Department of Drug Design and Pharmacology, University of Copenhagen, Universitetsparken 2, Copenhagen 2100, Denmark; b Biopharmaceuticals Research Unit, Novo Nordisk A/S, Novo Nordisk Park 1, 2760 Måløv, Denmark; c Biopharmaceuticals Research Unit, Novo Nordisk A/S, Life Science Park Road 29, Beijing 102206, People’s Republic of China

**Keywords:** IgG antibody, solution conformation, small-angle X-ray scattering (SAXS), structure modelling, shape clustering

## Abstract

IgG subclass-specific differences in biological function and *in vitro* stability are often referred to variations in the conformational flexibility, while this flexibility has rarely been characterized. Here, small-angle X-ray scattering data from IgG1, IgG2 and IgG4 antibodies, which were designed with identical variable regions, were thoroughly analysed by the ensemble optimization method. The extended analysis of the optimized ensembles through shape clustering reveals distinct subclass-specific conformational preferences, which provide new insights for understanding the variations in physical/chemical stability and biological function of therapeutic antibodies. Importantly, the way that specific differences in the linker region correlate with the solution structure of intact antibodies is revealed, thereby visualizing future potential for the rational design of antibodies with designated physicochemical properties and tailored effector functions. In addition, this advanced computational approach is applicable to other flexible multi-domain systems and extends the potential for investigating flexibility in solutions of macromolecules by small-angle X-ray scattering.

## Abstract 2

An extended analysis of structural ensembles obtained from small-angle X-ray scattering data reveals subclass-specific conformational preferences of IgG antibodies, which are largely determined by the hinge-region structure.

## 1. Introduction   

Immunoglobulin G (IgG) antibodies are the dominant antibodies in the human immune system and the IgG monoclonal antibodies (mAbs) are a major class of biopharmaceuticals with high antigen specificity and long half-lives. There are four human IgG subclasses, namely IgG1, IgG2, IgG3 and IgG4, which have been commonly described as flexible adaptor molecules with dual function, *i.e.* antigen binding (*via* the variable regions) and effector function (*via* the constant regions). Although the primary sequence in the constant regions is more than 90% identical, the IgG subclasses exhibit different effector functions, including complement activation and antibody-dependent cell-mediated cytotoxicity (Bruhns *et al.*, 2009 ▶; Brüggemann *et al.*, 1987 ▶). Interestingly, IgG subclasses with identical variable regions also exhibit different functional affinity to their antigens and differ in their ranking orders amongst different binding studies (Hubbard *et al.*, 2013 ▶; McCloskey *et al.*, 1996 ▶; Morelock *et al.*, 1994 ▶).

An IgG antibody consists of three mainly rigid regions (two Fab and one Fc domain) and two flexible linkers (hinge regions connecting the Fab and Fc domains), the linkers being expected to modulate the domain motion for immunological reactions (Roux *et al.*, 1997 ▶). Human IgG subclasses differ predominantly in their hinge-region sequence, length and disulfide bond structure (Liu & May, 2012 ▶), so solution conformations may differ depending on the nature of the antibody hinge region. Distinct subclass-specific characteristics can be identified in a study of immune-complex formation with bivalent haptens and the hinge-mediated flexibility of human IgG subclasses is reported ranking as IgG3 > IgG1 > IgG4 > IgG2 (Roux *et al.*, 1997 ▶). Only three crystal structures of intact IgG antibodies (human IgG1, murine IgG1 and murine IgG2a) have been determined (Harris *et al.*, 1992 ▶, 1998 ▶; Saphire *et al.*, 2001 ▶). The crystal structure of human IgG1 with a full-length hinge region reveals a strikingly asymmetric conformation somewhere between a T- and a Y-shape. The extended hinge region of the two heavy chains also shows variable torsion angles, which suggests a considerable degree of flexibility (Saphire *et al.*, 2002 ▶). Altogether, antibodies in solution exhibit significant hinge-mediated structural flexibility, which might influence antigen recognition, effector functions, and general solution and stability properties such as self-association, precipitation, viscosity, fragmentation *etc*. Therefore, in-depth characterization of the solution structure of intact antibodies under physiological and formulation-relevant conditions could facilitate antibody engineering and isotype selection in the development of antibody therapeutics.

Small-angle X-ray scattering (SAXS) enables investigation of the solution structure of macromolecules under such conditions (Mertens & Svergun, 2010 ▶). Abe *et al.* (2010 ▶) reported an asymmetric solution structure of mouse–human chimeric IgG4 by constrained scattering modelling, in which the Fc region is masked by one Fab region. The global *ab initio* envelopes of four murine IgG subclasses with identical variable regions further revealed subclass-dependent average domain orientations (Eryilmaz *et al.*, 2013 ▶). However, the static average conformations revealed by *ab initio* or constrained scattering modelling reported in those two studies do not provide a basis for investigating the flexibility of the IgGs. A few recent studies have partially addressed the structural flexibility of antibodies in solution. Clark *et al.* (2013 ▶) utilized neutron scattering data to demonstrate potential, but not optimized, structural ensembles of an IgG2 mAb. Lilyestrom *et al.* (2012 ▶) reported the bimodal size distribution of IgG1 mAb structures and, by selecting minimum ensembles, showed the presence of an open and closed conformation.

Here, we adapt the full ensemble optimization method (EOM), enabling us to optimize the structural ensembles with flexible size and adjustable frequency of each structure (Tria *et al.*, 2013 ▶; Petoukhov *et al.*, 2012 ▶), closely reflecting the solution behaviour of the IgGs. This approach is applied to SAXS data from humanized IgG1, IgG2 and IgG4 mAbs with identical variable regions. We markedly expand the typical analysis of the selected ensembles by clustering the structures of the optimized ensembles based on their overall shapes. Our in-depth analysis of these ensembles reveals distinct conformational preferences and hinge-mediated segmental flexibility of the three IgG subclasses. Hence, we report for the first time a detailed comparative structural study, including the varied solution conformations exhibited by humanized IgG subclasses, and provide new insights into how solution conformation affects biological function and physicochemical properties.

## 2. Materials and methods   

### 2.1. Materials   

Humanized monoclonal antibodies (IgG1, IgG2 and IgG4 S241P) with identical light chains and identical variable regions in the heavy chains were produced as described previously (Tian *et al.*, 2014 ▶). Protein samples were exchanged into 50 m*M* sodium phosphate (pH 7.4) containing 100 m*M* NaCl and further concentrated to approximately 12 mg ml^−1^ with 30 kDa MWCO Amicon Ultra-4 centrifugal ultrafiltration devices (Millipore). The flow-through buffer for each sample was collected respectively and stored at 4°C together with protein stock until measurement.

### 2.2. SAXS data collection and primary data analysis   

Prior to the data collection, the protein stock was centrifuged at 13 000 rev min^−1^ for 10 min and then diluted into four samples of approximately 1, 2, 6 and 12 mg ml^−1^. The protein concentrations were verified using a NanoDrop ND-1000 spectrophotometer (NanoDrop Technologies Inc, Wilmington, Delaware, USA). Data collection was performed at the EMBL beamline X33 (Blanchet *et al.*, 2012 ▶, Roessle *et al.*, 2007 ▶) on the DORIS III storage ring (DESY, Hamburg, Germany). Scattering intensity was measured as a function of momentum transfer [defined as *q* = 4πsin(θ)/λ, where 2θ is the scattering angle and λ is the X-ray wavelength; λ = 1.5 Å]. Samples were loaded into a flow cell cooled to 8°C, using a robotic sample changer (Round *et al.*, 2008 ▶), and exposed for 8 × 15 s, and the data were checked for radiation damage before averaging. The flow-through buffers, collected during buffer exchange, were used for background subtraction and the primary data analyses were done using *PRIMUS* (Konarev *et al.*, 2003 ▶), in which *GNOM* (Svergun, 1992 ▶) was subsequently used to generate the pair distance distribution functions. Radius of gyration (*R*_g_) and the maximal dimension (*D*_max_) were then derived from the SAXS data. The correlation length (*l*_c_) was determined using *SCÅTTER* (Rambo & Tainer, 2011 ▶), and the volume of correlation (*V*_c_) was calculated by *V*_P_/2π*l*_c_, where *V*_P_ is the Porod volume (Rambo & Tainer, 2013 ▶). The molecular mass was then directly determined from its linear relationship with *Q*_*R*_ in a log–log plot (where *Q*_R_ is defined as the ratio of *V* ^2^_c_/*R*_g_; Rambo & Tainer, 2013 ▶). The apparent particle density of each molecule was further calculated by dividing the Porod volume into the molecular mass (Rambo & Tainer, 2011 ▶).

### 2.3. Homology modelling   

The primary sequence of the humanized IgG1 in this study was designed with the same constant region as crystallized IgG1 *b*12 (Saphire *et al.*, 2001 ▶) (PDB entry 1hzh), thus the Fc domain was extracted directly from the crystal structure as a template for IgG1. The protein sequence of the IgG1 Fab domain was most similar to that of the isolated Fab crystallized in complex with epidermal growth factor (Li *et al.*, 2008 ▶) (PDB entry 3b2u). Based on identical constant regions, the most similar variable region of the heavy chain and 61% sequence identity in the variable region of the light chain, 3b2u was selected as a template for the full IgG1 Fab domain in order to maintain integrity. The Fc domain structure from 1hzh and the Fab domain structure from 3b2u were also identified as the best templates for IgG2 and IgG4. Homology models of Fab and Fc domains for each IgG subclass were built using *SWISS-MODEL Workspace* in *Project Mode* (Arnold *et al.*, 2006 ▶). The glycan was added onto each C_H_2 domain with nine monosaccharides, according to the high-resolution crystal structure (PDB entry 1hzh). The theoretical SAXS curve of the crystal structure of the full-length IgG1 (PDB entry 1hzh) was also calculated using *CRYSOL* (Svergun *et al.*, 1995 ▶) with default parameters.

### 2.4. Rigid-body modelling   

**Figure 1:**
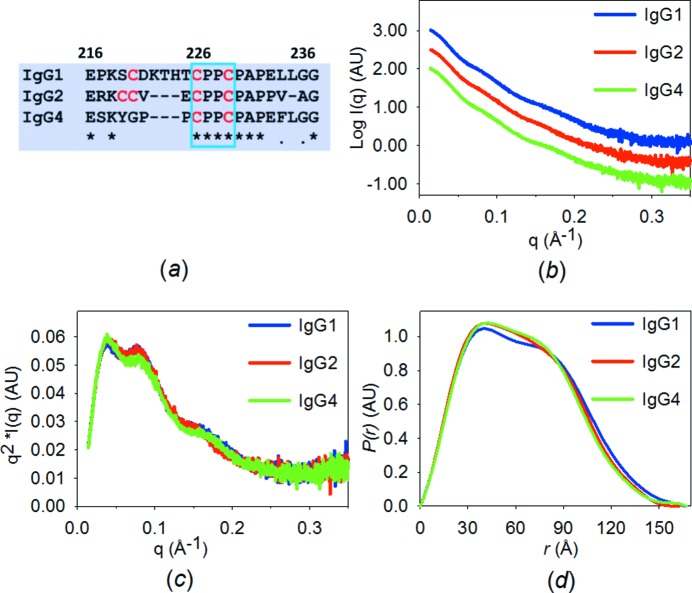
Solution scattering data. (*a*) A comparison of the primary sequence of hinge regions in the three IgG subclasses. The locations of the small rigid bodies are highlighted within the small blue box. (*b*) Scattering intensity plot and (*c*) Kratky plot of SAXS data collected on the IgGs in 50 m*M* Na phosphate, 100 m*M* NaCl, pH 7.4. IgG1 (blue), IgG2 (red) and IgG4 (green). (*d*) The pair distance distribution functions from the indirect Fourier transformation of the scattering intensity.

Rigid-body modelling of IgG1 was performed using *CORAL* (Petoukhov & Svergun, 2005 ▶) with default parameters. The Fab and Fc homology models were connected by flexible linkers according to the primary sequence of the hinge region (corresponding to the residues/linkers shown in Fig. 1 ▶ *a*). In order to simulate the disulfide bonds, we included contact conditions with a maximum distance of 5.6 Å between the C^α^ atoms of the cysteines in the middle hinge region.

### 2.5. Ensemble optimization   

The Fab and Fc domains were treated as rigid bodies using the homology models. In addition, two small hinge fragments with the sequence CPPC were extracted from the crystal structure (PDB entry 1hzh) and treated as one rigid entity to simulate the disulfide bonds in the central hinge region. The four rigid bodies were connected by flexible linkers according to the remaining amino acids of the hinge region (Figs. 1 ▶ *a* and 2 ▶ *i*). Using this approach, pools containing 10 000 structure models for each IgG subclass were generated using *RANCH* from the *EOM* package (Petoukhov *et al.*, 2012 ▶; Bernadó *et al.*, 2007 ▶). The optimized ensemble was selected using the genetic algorithm within *GAJOE* (Bernadó *et al.*, 2007 ▶). 1000 generations were applied and a flexible ensemble size was allowed. In addition, each structure model has a variable frequency in the conformational ensemble. The genetic algorithm process was repeated 100 times. Both *R*_g_ and *D*_max_ distributions were calculated from all the optimized ensembles for each individual IgG subclass.

### 2.6. Analysis of optimized ensembles   

In this study, we went further to extract conformational information for each IgG subclass by performing a particle shape-based clustering of all structures obtained from ten optimized ensembles for each of the IgGs (*i.e.* from ten random runs of *GAJOE*). The number of clusters was determined using *DAMCLUST* (Petoukhov *et al.*, 2012 ▶). Firstly, the normalized spatial discrepancy (NSD) between each pair of models was calculated by *SUPCOMB* (Kozin & Svergun, 2001 ▶), based on the backbone of the structure models. Secondly, the NSD matrix was used for hierarchical cluster analysis, where the two nearest clusters/structures were merged to form a single cluster at each stage (Kelley *et al.*, 1996 ▶). At the same time, the spread (Sp) of each cluster was calculated by
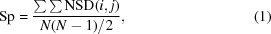
where *N* is the number of structures in the corresponding cluster. The spreads of all the clusters were then averaged and normalized to lie within the range 1 to (*M*−1), where *M* is the total number of structures. Finally, a penalty function, which is defined as the summation of the normalized average spread and the number of clusters, was applied to seek a cut-off value, where the number of clusters is minimized but the structures in each cluster are maintained as similar as possible. The overall occurrence and average *D*_max_ of the obtained cluster were then calculated by summing the frequency of each structure model in the corresponding ensemble. All the structure models in the same cluster were superimposed by *SUPCOMB*. The Fc orientations of the structure models in each cluster were checked manually and the probability of the Fc region on each rigid domain was calculated.

## 3. Results   

### 3.1. IgG flexibility and subclass differences are directly observable in the SAXS data   

We apply our studies to three humanized IgG subclasses designed with an identical primary sequence in the entire region of the light chains and the variable region of the heavy chains. The largest difference between the IgG subclasses is hence the hinge region, enabling exclusive comparison of subclass-related solution behaviour (Fig. 1 ▶ *a*).

We have previously reported that the solution state of the individual IgGs is unaffected by pH changes within the range pH 5.0–8.5 (Tian *et al.*, 2014 ▶; Fig. S1 in the supporting information), which also demonstrates the robustness and consistency of the SAXS measurements. However, subtle differences were observed between IgG subclasses (Figs. 1 ▶ *b* and 1 ▶ *c*), despite the small sequence differences. These differences are also reflected by the pair distance distribution functions obtained from the indirect Fourier transformation of the scattering data, which show that IgG1 has slightly larger dimensions (Fig. 1 ▶ *d*) and accordingly exhibits the largest values for the radius of gyration (*R*_g_) and volume of correlation (*V*_c_) (Table 1 ▶). The molecular masses determined using the ratio *Q*_R_, which is contrast- and concentration-independent (Rambo & Tainer, 2011 ▶), are in accordance with the theoretical values, and the subtle differences between the SAXS curves reflect the conformational differences in solution.

IgG molecules in solution exhibit hinge-mediated differences in flexibility (Roux *et al.*, 1997 ▶). The three peaks observable in the Kratky plots confirm that IgG molecules are multi-domain proteins with flexible linkers (Glatter & Kratky, 1982 ▶) (Fig. 1 ▶ *c*). The calculated particle volumes (see Table 1 ▶) are all significantly larger than *e.g.* that calculated from the IgG1 crystal structure (PDB entry 1hzh). Also, the corresponding average apparent particle densities are all significantly lower than the mean empirical protein density (approximately 1.35 g cm^−3^; Quillin & Matthews, 2000 ▶), hence each domain is, on average, present in a lower average density, leading to the conclusion that the increased volumes imply extensively flexible IgG solution conformations (Rambo & Tainer, 2011 ▶).

### 3.2. Conformational ensembles describe the distinct solution state adapted by individual IgG subclasses   

**Figure 2:**
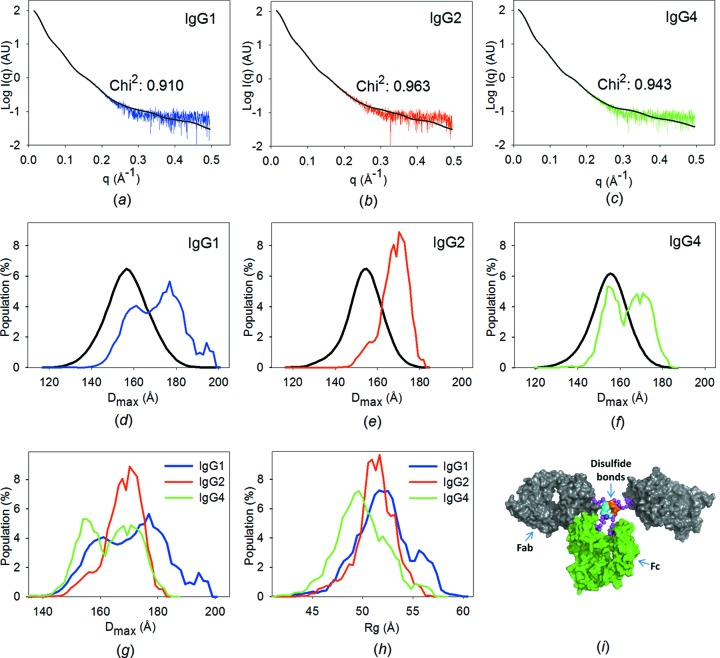
Results of ensemble optimization. (*a*)–(*c*) Fits between the calculated scattering curve from the best ensemble (coloured, selected by EOM) and the experimental data (black). (*d*)–(*f*) *D*_max_ distributions of the conformers in the pool (black) and the optimized ensembles (coloured). (*g*) Comparison of the *D*_max_ and (*h*) of the *R*_g_ distributions in the optimized ensembles of the three IgG subclasses. (*i*) A representative structural model of IgG1 generated by *RANCH*.

The observed lowered average particle density and increased volumes together infer structural flexibility. Accordingly, the experimental scattering data could not be fitted by theoretical scattering from the known crystal structure (PDB entry 1hzh) (Fig. S2*a*). Although an improved fit was obtained using rigid-body modelling, the theoretical scattering of this single conformation still yields discrepancies with the experimental data (Fig. S2*b*). Rather, the SAXS data must be fitted by ensembles of structures with variable frequency. Using the genetic algorithm in *EOM* (Bernadó *et al.*, 2007 ▶; Petoukhov *et al.*, 2012 ▶; Tria *et al.*, 2013 ▶), ensembles of data curves were selected from large pools. The pools included the calculated scattering profiles for 10 000 IgG structures for each subclass, generated with the corresponding hinge region in random conformations. This procedure yields excellent fits with very low standard deviations [χ^2^ = 0.944 ± 0.018 (IgG1), 1.027 ± 0.021 (IgG2) and 0.978 ± 0.017 (IgG4), averaged over 100 repetitions], which suggests that the selected ensembles are reproducible. The best fits between the experimental SAXS data and theoretical SAXS data based on the ensembles are shown in Figs. 2 ▶(*a*)–2 ▶(*c*), and the high-quality fits can be visually emphasized in a Kratky plot (exemplified in Fig. S2).

The robustness of the solutions reflecting the conformational diversity was assessed by several means. Firstly, we calculated distributions of the maximal dimension (*D*_max_) for the models from 100 optimized ensembles, and in all cases the three IgG subclasses exhibit highly consistent *D*_max_ distributions (Figs. S3*a*–S3*c*). Secondly, we varied the number of generations applied in the genetic algorithm, and the χ^2^ values decrease only slightly when increasing the generations (Fig. S3*d*). In addition, ensemble optimization was tested using a smaller angular range (0–0.3 Å^−1^), yielding distribution profiles (Fig. S4) consistent with those obtained from the full angular range. These tests hence assure the reliability of the selected ensembles. When analysing the selected ensembles, it is evident that the *R*_g_ and *D*_max_ distributions of the selected models have larger values than the average distribution of the starting pool (Figs. 2 ▶ *d*–2 ▶ *f*, Fig. S5, Table S1), indicating a preference for overall extended conformations in solution. In addition, the distribution profiles from the ensembles differ significantly for the three IgG subclasses (Fig. 2 ▶ *g*). In this analysis, the internal flexibility of the individual Fab and Fc fragments is considered negligible (Fig. S6), so the Fab and Fc fragments are assumed to be rigid entities and the overall particle dimension should be described mainly by Fab–Fab and Fab–Fc flexibility, *i.e.* hinge-region flexibility. IgG2 has the narrowest distribution profile of the three IgGs, reflecting a relatively high rigidity with a fairly stable particle size. IgG1 has the broadest *D*_max_ distribution with a shift towards larger particle sizes, correlating with the longer hinge region (Fig. 1 ▶ *a*). The length of the IgG4 hinge region is comparable with that of IgG2. Interestingly, however, IgG4 exhibits an intermediate *D*_max_ distribution, while the particle dimensions are shifted towards more compact conformations. The *R*_g_ distributions were also calculated from 100 optimized ensembles. These distribution profiles show the same patterns when comparing the three IgG subclasses, which further verifies their differential solution conformations (Fig. 2 ▶ *h*) and provides a third evaluation of the robustness of the investigated parameters.

### 3.3. Subclass-specific shape clustering of the selected structures   

**Figure 3:**
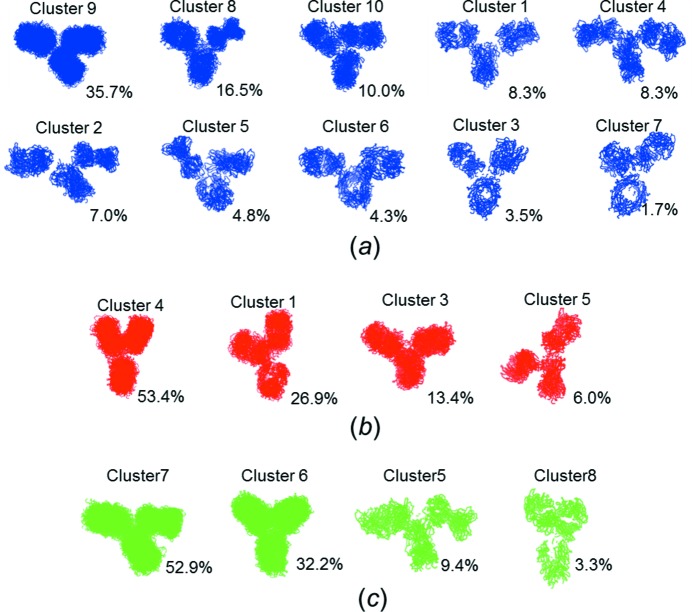
Superimposed structures from the conformational subsets. The clusters for (*a*) IgG1, (*b*) IgG2 and (*c*) IgG4 are shown in decreasing order of overall occurrence, which is noted in the lower right-hand corner of each plot. The domains pointing downwards are the regions mostly occupied by the Fc domain.

For intrinsically disordered proteins, EOM is primarily applied to evaluate the degree of flexibility and the level of compactness. For flexible multi-domain proteins, further assessment of *e.g.* inter-domain contacts is possible (Bernadó *et al.*, 2007 ▶). There is ambiguity in the individual structures from the selected ensemble, so analysis of the individual structure is not reliable. In this study, the structural pools were generated based on knowledge of the Fab and Fc fragments and the hinge linkers, representing the expected conformations of intact molecules, so we examined the trends of the conformational sampling with respect to segmental flexibility. To avoid interpretation of individual structural models, we investigated the total pool of structures from ten random optimized ensembles, which can statistically reduce the variation in the EOM results. We further clustered these structures based on size and shape. The number of clusters was determined by a penalty function, where the minimum penalty value was objectively selected as the cut-off level, ensuring that the most similar conformations were as highly populated as possible. We thoroughly inspected all the clusters and observed that the structures in some clusters had different Fab/Fc orientations. It is well known that IgG Fab and Fc fragments have similar immunoglobulin folds and overall dimensions, and the Fc fragments have only a slightly higher excluded volume. This also means that two structures which can be superimposed based on their overall shapes, but with their Fab and Fc domains in diverging positions, will have comparable theoretical scattering profiles. Our procedure may result in ambiguous assignment of Fab/Fc position, and if a given structure is present at a low frequency in the selected ensemble, it would yield a negligible difference in the fit between the experimental and theoretical SAXS data. In order to identify the most probable orientation (*i.e.* distinction between Fab and Fc) in each cluster, we thus calculated the occupancy of Fab or Fc within each rigid-body region of the superimposed structures. The resulting final structural clusters for each of the three IgG subclasses are shown in Fig. 3 ▶. In the orientation shown, the lower domain corresponds to the most probable Fc region. The percentage of structures with their Fc domain in this orientation was calculated (Table 2 ▶) and should be related to a random distribution, in which case the probability would be about 33% (*i.e.* the Fc domain is located equally often in each of the three domains and is thus completely ambiguous). In addition, we qualitatively describe the conformational ensembles by denoting those structures with ‘open’ arms and a larger Fab–Fab distance as a T-shaped conformation, and those with ‘closed’ arms and a smaller Fab–Fab distance as a Y-shaped conformation.

The conformation of IgG1 is described by a higher number of clusters than IgG2 and IgG4 (Fig. 3 ▶ and Table 2 ▶). About one third of the IgG1 structures are in the major cluster (cluster 9) with intermediate *D*_max_ values, while several minor clusters are distributed with overlapping *D*_max_ values covering the entire range, underlining the high flexibility of IgG1. No, or only minor, ambiguity can be observed in the Fc orientation (Table 2 ▶), so the solution conformation of IgG1 can be elaborated as follows.

(i) Clusters 1, 2, 4 and 6 exhibit a T-shape with large Fab–Fab distances. These conformational species are in accordance with the ‘open’ conformation of IgG1 reported by Lilyestrom *et al.* (2012 ▶). In comparison, clusters 2 and 4 have a similar Fab–Fc pair on one side in close proximity, while the second Fab arm of cluster 2 is further from the Fc region.

(ii) We also observed different kinds of IgG1 Y-shapes (clusters 3, 5, 7, 8 and 10). Clusters 5 and 10 represent relatively compact conformations with lower *D*_max_ (Table 2 ▶), in which the Fab and Fc fragments either bend to one side or are asymmetrically arranged.

(iii) The dominant cluster 9 is in an intermediate conformational state between T- and Y-shaped.

**Figure 4:**
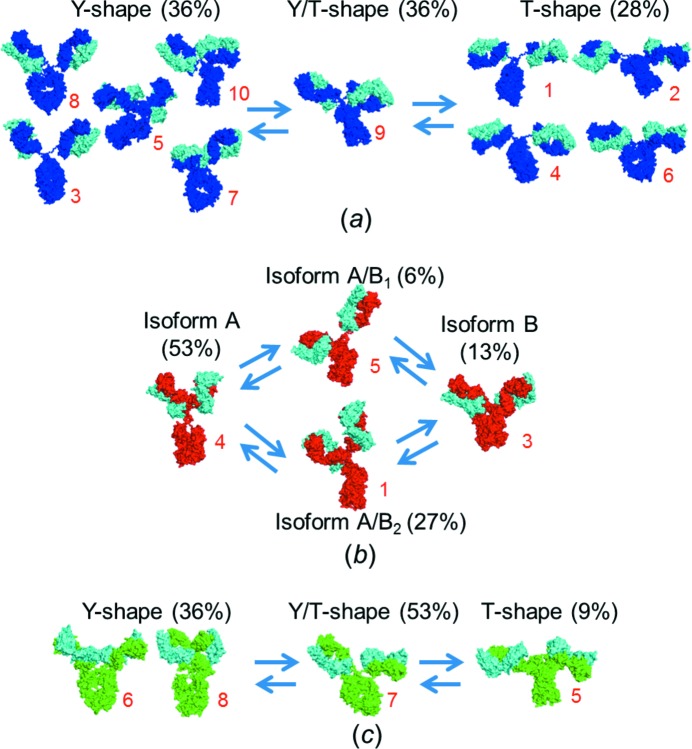
Putative conformational equilibria of (*a*) IgG1, (*b*) IgG2 and (*c*) IgG4. Representative structures are selected from the corresponding clusters. The number of the cluster is noted in the lower right-hand corner in red. The structure types and their overall percentage in the optimized structural pool are noted above each group.

Altogether, IgG1 accommodates diverse conformations from T-shape to Y-shape. The *D*_max_ of IgG1 increases in the order Y-shape < Y/T-shape < T-shape (Table 2 ▶ and Fig. 4 ▶ *a*), and it presents a unique distribution profile with two major peaks (Fig. 2 ▶ *g*), correlating with the Y- and T-shapes.

Both IgG2 and IgG4 cluster into only four distinct overall conformations (Fig. 3 ▶). Interestingly, both subclasses have one very dominant cluster, representing more than half of the selected structural models. Therefore, only a few alternative overall conformations of IgG2 and IgG4 exist in solution, in contrast with the consecutive conformational shifts observed for IgG1. The dominant cluster (cluster 4) of IgG2 exhibits a typical Y-shape and has the Fc domain unambiguously positioned in the downward position (Fig. 3 ▶), while clusters 1 and 3 (27% and 13% of the selected structures, respectively) deviate slightly from the Y-shape, and only the minimally occurring cluster 5 exhibits an ‘open’ conformation with one Fab arm swinging down. The IgG4 solution state is almost fully described by two dominant clusters (clusters 6 and 7). Notably, however, both class assignments are ambiguous with respect to Fc orientation. In cluster 5 both of the IgG4 Fab arms are in close proximity to the Fc region. The other conformational extreme (cluster 8), with a very close proximity between the two Fab arms, is only represented in low numbers. The summations of the most dominant clusters of IgG2 and IgG4, as shown in Fig. 3 ▶, cover more than 99% and 97% of the selected structures, respectively, so the other clusters contain rare structures for both subclasses. However, IgG4 exhibits larger observable differences between the clusters and broader *D*_max_ distribution profiles (Fig. 2 ▶ *g*), leading to the conclusion that IgG4 is more flexible than IgG2. In conclusion, the flexibility can be described as IgG1 > IgG4 > IgG2.

## 4. Discussion   

### 4.1. Structural integrity of individual IgG subclasses   

The three IgG antibodies of the present study exhibit no noticeable structural changes in a pH range of 5.0–8.5 (Tian *et al.*, 2014 ▶). Accordingly, Lilyestrom *et al.* (2012 ▶) reported that the conformational equilibrium of two IgG1 variants is un­affected by a broad range of co-solutes (ion types and ionotropic strength), the only exception being arginine chloride, which favours an ‘open’ antibody conformation. We also observed in our previous studies (Tian *et al.*, 2014 ▶) that variation of the formulation excipients (NaCl and sucrose) does not result in detectable changes in solution conformation. In conclusion, the structural distribution of individual IgG subclasses is stable under the investigated experimental conditions. In contrast, we observe significant structural differences, directly observable from distinct differences in the raw SAXS data curves, between different IgG subclasses. Since we are working with engineered antibodies exhibiting identical variable regions, we conclude that the observed differences originate from their inherent hinge-region properties, including the hinge-region length and the different patterns and numbers of intramolecular disulfide bridges.

As in previous studies (Clark *et al.*, 2013 ▶; Lilyestrom *et al.*, 2012 ▶), we conclude that IgG antibodies are flexible in solution. Importantly, we have revealed very clear differences in the structural preferences of the IgG subclasses. Clearly, IgG4 and particularly IgG2 adapt to fewer overall conformations in solution, while IgG1 reveals a continuum of conformations around the preferred intermediate Y-shape. To the best of our knowledge, such significant subclass-specific differences have not been characterized before. The importance of these studies includes, firstly, the possibility of linking the different structural behaviours with differences in the stability and biological function of IgG subclasses and, secondly, of using this structural insight in the future design and development of therapeutic antibodies.

### 4.2. Implications for biological function   

A large number of reports have demonstrated the differences between the four IgG subclasses on complement fixing and FcγR binding (Brüggemann *et al.*, 1987 ▶; Bruhns *et al.*, 2009 ▶), while the relationship between antibody flexibility and effector function remains unclear. Dall’Acqua *et al.* (2006 ▶) reported that decreasing the rigidity of the middle hinge region negatively affects C1q binding. They also demonstrated that specific modifications of the upper hinge region are able to enhance complement fixation, but they did not, at that time, have a tool such as the one presented here to characterize the solution structure, so it was difficult to conclude to what extent the alteration in flexibility influences these effector functions. Here, we reveal how IgG1, IgG2 and IgG4 adapt to distinct and complementary preferred conformational ensembles, which might contribute to the differential effector functions. As an example, IgG4 adapts to a preferred Y/T-shape, which implies a possible steric hindrance on one C1q binding site when the antibody adapts to this conformation (which we observe in almost 53% of all cases under the experimental conditions investigated). However, Xu *et al.* (1994 ▶) reported that a single mutation (S331P) in the C_H_2 domain of IgG4 can restore approximately 50% of complement-binding ability compared with IgG1. There is thus a complex relationship between flexibility and activity. The primary structure and the preferred conformation or flexibility of antibodies might have collective effects on the effector functions, where the primary structure is expected to play the determinant role, but further studies are needed to elucidate the importance of the different parameters.

Segmental flexibility was previously suggested to correlate with the differential antigen-binding property of IgG subclasses with identical variable regions (Morelock *et al.*, 1994 ▶), although the ranking order of the flexibility was not always consistent with the functional affinity (Hubbard *et al.*, 2013 ▶; McCloskey *et al.*, 1996 ▶). In another study, the binding affinity to monovalent antigen was proved to be affected by the constant region of the antibody (Torres *et al.*, 2007 ▶), implying that the paratope site could be manipulated by altering the microenvironment in order to induce a better ‘fit’ to the antigen. In the light of our study, the preference for either Y- or T-conformations reflects different intramolecular Fab–Fab or Fab–Fc interactions, which potentially offers additional insight into the observed differences (Torres *et al.*, 2007 ▶). Hence, flexibility is not the only factor accounting for avidity during antigen binding. Rather, we speculate that conformational preference is important in understanding the differences in affinity amongst IgG subclasses. Thus, it seems plausible that the affinity and specificity will be influenced by the very different conformational subsets observed in our study.

### 4.3. Implications for physical and chemical properties   

It is of interest to investigate whether the current observations of the very distinct conformational preferences of the IgG subclasses can explain some of the well known subclass-specific differences in solution stability. As an example, it is well known that IgG2 and IgG4 are more prone to aggregation than IgG1 (Ishikawa *et al.*, 2010 ▶). We have demonstrated above that IgG2 has a dominant Y-shape and IgG4 has alternating conformational shifts between a Y-shape and a preferred Y/T-shape in solution, while IgG1 reveals a much greater flexibility, accommodating almost one third of T-shaped conformations. A number of aggregation-prone motifs of IgG1 have been identified (Chennamsetty *et al.*, 2009 ▶), most of which are concentrated around the lower hinge region. In addition, these motifs are largely preserved in the primary sequences of all IgG subclasses. Thus, we suggest that the T-shape might shield the aggregation-prone motifs (Chennamsetty *et al.*, 2009 ▶) of IgG1 and improve its physical stability. A second possible link between the observed structural ensembles and antibody stability is based on the reported differences in fragmentation for IgG subclasses. IgG1 is more susceptible to non-enzymatic cleavage (Ishikawa *et al.*, 2010 ▶). As several active cleavage sites have been identified in the upper hinge region of IgG1 (Vlasak & Ionescu, 2011 ▶), we anticipate that the flexibility and solvent exposure of this region also influence fragmentation. However, the overall fragmentation susceptibility is likely to be determined by collective effects of both primary and tertiary structure.

Very few crystal structures of intact antibodies have been determined, which can be explained by the observed heterogeneity of the solution structures of the three IgG subclasses in our study. Interestingly, the crystal structure of human IgG1 *b*12 (PDB entry 1hzh) shows a similar asymmetric conformation (Y/T-shaped) as the dominant solution conformation of the present IgG1 (and IgG4), which might be induced by specific Fab–Fc interactions. Thus, it may be possible to crystallize the intact antibody if an ideal solvent condition can be found to increase the homogeneity. As has been demonstrated here, SAXS is a powerful tool for monitoring these structural equilibria, and since SAXS is further highly suited to extensive screening of different solvent conditions, we suggest that it may be used to identify experimental conditions suitable for the crystallization of flexible molecules, such as antibodies. It would suffice to perform the calculation of the *D*_max_ distribution, hence avoiding the time-consuming analysis of selected conformations that we have performed here. Rather, the identification of experimental conditions resulting in a narrow *D*_max_ distribution would indicate potential crystallization-prone conditions.

### 4.4. Linker-region distinctions define the conformational preferences of IgG1, IgG2 and IgG4   

As mentioned above, IgG1 reveals the highest degree of flexibility, adapting to both Y-, intermediate and T-shapes (Fig. 4 ▶ *a*). This is in good accordance with the long hinge region of this subclass. In contrast, the primary and secondary structure of IgG2 reveals a ‘rod-like’ hinge region with four possible disulfide bonds, and the Fab–Fab flexibility is further restricted by the short upper hinge. Indeed, we observe that IgG2 has a relatively narrow *D*_max_ distribution, and although diverse conformational subsets can be identified with overlapping *D*_max_ values, more than half of the structures exhibit a well defined Y-shaped conformation. We propose that this cluster (cluster 4 in Fig. 3 ▶) corresponds to the major IgG2 disulfide isoform A (IgG2-A), as characterized by Dillon *et al.* (2008 ▶) (Fig. 4 ▶ *b*). The corresponding IgG2-B isoform, in which both Fab arms are covalently linked by the two upper hinge cysteine residues (Dillon *et al.*, 2008 ▶), very likely corresponds to cluster 3 of IgG2 (Fig. 3 ▶), with close proximity between the upper and lower parts of the structure, but only occupied by approximately one in eight IgG2 molecules (Fig. 3 ▶). Martinez *et al.* (2008 ▶) reported the presence of two structural intermediates (IgG2-A/B_1_ and IgG2-A/B_2_), which we suggest correspond to clusters 5 and 1, respectively (Fig. 3 ▶, IgG2). IgG2-A/B_1_ has an intra-chain disulfide bond on one side of the hinge region, which can increase the Fab–Fab flexibility. In addition, one Fab arm can be pulled down by the formation of a disulfide linkage with the hinge region, thus corresponding to the observed ‘open’ Fab arms in cluster 5 (Fig. 3 ▶). IgG2-A/B_2_ has a classical disulfide linkage on one side, resulting in restricted Fab–Fab flexibility. Thus, when the Fab arm with a Fab–hinge linkage swings to one side, the other Fab arm will follow, resulting in an asymmetric solution conformation in the IgG2-A/B_2_ isoform, in good accordance with our observations in cluster 1. Hence, the directly observed structural preferences of IgG2, characterized by thorough ensemble modelling of the SAXS data, are directly in accordance with the reported presence of different isoforms, based on variations in the number of disulfide bonds. In addition, the biophysical characterization of redox-enriched isoforms using size-exclusion chromatography and analytical ultracentrifugation demonstrates that IgG2-A has an increased hydrodynamic radius relative to IgG2-B (Dillon *et al.*, 2008 ▶). Hence, overall, our findings for IgG2 suggest a distinct interpretation of the structural changes linked to the variation in disulfide distribution. The full suggested conformations of IgG2 with respect to the corresponding disulfide isoforms are shown in Fig. 4 ▶(*b*).

IgG4, like IgG1, has two major peaks in the *D*_max_ distribution profile of the selected ensembles, but both peaks are shifted towards relatively low values of *D*_max_. This reflects the shorter linker region of IgG4. The two major peaks in the *D*_max_ distribution correlate with the two dominant IgG4 conformational clusters 6 and 7, occurring with a bias towards cluster 7 (asymmetric open Y/T-shape; see IgG4 in Fig. 3 ▶). Cluster 7 has features reminiscent of those in the corresponding Y/T-shaped cluster 9 of IgG1. Hence, both IgG1 and IgG4 reveal an equilibrium between Y- and T-shapes (Fig. 4 ▶ *c*), only with a significantly reduced degree of general flexibility for IgG4. IgG4 is preferentially kept in the intermediate Y/T-conformation, with only a minor part in the T-shaped structure (about 10%). Noticeably, the commonly used S241P IgG4 variant analysed in this study is known to have a lower propensity for half-antibody generation (Angal *et al.*, 1993 ▶). The mutant proline residue is expected to confer further rigidity on the middle hinge region, thereby shifting the conformational equilibrium towards the Y-shape. It has been reported that the Fab arms can stabilize the covalent disulfide isomer of IgG4 (Rispens *et al.*, 2011 ▶), and the Y-shape is likely to play an important role by decreasing the accessibility of its hinge region. In the T-conformation the disulfide bonds are prone to be reduced, which would further facilitate the generation of half-antibodies. We suggest that the reason for the increased S241P IgG4 solution integrity is a shift in the preferred conformation from T- to predominantly Y/T- and Y-shapes.

## 5. Conclusions   

We have presented a thorough analysis of the flexibility of three IgG subclasses in solution by clustering the structural models from optimized ensembles based on the shape of the macromolecules. The subclass-specific conformational preferences provide potential explanations for their differences in biological function and physicochemical properties. Importantly, we have shown that specific linker-region differences correlate with observed structural differences. It is thus possible to use this insight for the rational design of antibodies, not only with increased or decreased flexibility, but notably also with a particular preference for a subset of conformations. SAXS-based ensemble analysis is uniquely suited to the study of such designed modifications of conformational ensembles. Hence, our study provides a first example of a novel tool for the future design of improved therapeutic antibodies.

## Supplementary Material

Supporting information file. DOI: 10.1107/S205225251402209X/tj5007sup1.pdf

## Figures and Tables

**Table 1:** Data-collection and scattering-derived parameters

	IgG1	IgG2	IgG4
Data-collection parameters (for all IgGs)			
Instrument	EMBL X33, DORISIII (DESY, Hamburg), Pilatus 1M detector		
Beam size at the detector (mm)	2 0.6		
Wavelength ()	1.5		
*q* range (^1^)	0.0090.500		
Exposure time (s)	15		
Concentration range (mgml^1^)	112		
Temperature (K)	281		

Structural parameters			
*I*(0) (cm^1^) (from Guinier)	0.1220.001	0.1180.001	0.1180.001
*R*_g_ () (from Guinier)	49.40.38	47.60.48	47.60.49
*I*(0) (cm^1^) [from *P*(*r*)]	0.124	0.120	0.119
*R*_g_ () [from *P*(*r*)]	51.0	49.4	49.0
*D*_max_ ()	162	162	158
Porod volume estimate (^3^)	250720	239520	248080
Dry volume (^3^)†	176829	176380	176434

Molecular-mass determination			
Correlation length ()	0.12	0.13	0.13
Volume of correlation (^2^)	939	908	910
*Q*_R_ (^3^)	17857	17338	17397
Molecular mass *M*_r_ (Dalton) (from *Q*_R_)	145000	141000	141000
Corresponding particle density (gcm^3^)	0.96	0.97	0.95
Monomeric *M*_r_ † (Dalton)	145000	145000	145000

Software employed (for all IgGs)			
Primary data reduction	*PRIMUS*		
Data processing	*PRIMUS*, *SCTTER*		
Homology modelling	*SWISS-MODEL*		
Rigid-body modelling	*CORAL*		
Ensemble modelling	*EOM*		
Shape clustering	*DAMCLUST*		
Computation of model intensities	*CRYSOL*		
Three-dimensional graphics representations	*PyMOL*		

† Calculated from the sequence.

**Table 2:** Shape clustering of the structures from ten optimized ensembles using *DAMCLUST*

Cluster	Number of PDB entries	Total frequency	*D*_max_ ()	Overall occurrence (%)†	Maximum probability of Fc orientation (%)‡
IgG1					
1	3	19	191.64.2	8.3	89
2	4	16	193.87.1	7.0	100
3	2	8	164.42.9	3.5	100
4	3	19	181.66.0	8.3	100
5	3	11	154.45.3	4.8	100
6	3	10	173.73.8	4.3	80
7	2	4	165.62.9	1.7	100
8	10	38	167.86.3	16.5	89
9	28	82	170.99.1	35.7	78
10	6	23	148.73.3	10.0	78
Total	64	230			
		
IgG2					
1	10	72	171.97.0	26.9	43
2	1	1	155.20.0	0.4	
3	9	36	167.37.3	13.4	36
4	21	143	168.66.7	53.4	74
5	4	16	172.54.1	6.0	100
Total	45	268			
		
IgG4					
1	1	1	140.00.0	0.4	
2	1	2	181.30.0	0.7	
3	1	1	139.70.0	0.4	
4	1	2	152.50.0	0.7	
5	3	26	175.36.8	9.4	100
6	25	89	158.08.3	32.2	44
7	35	146	165.08.1	52.9	47
8	2	9	162.08.0	3.3	100
Total	69	276			

† The overall occurrence is the percentage of the overall frequency of each cluster derived from ten optimized ensembles. The average ^2^ values of the ten optimized ensembles are 0.9480.02 (IgG1), 1.0250.02 (IgG2) and 0.9840.02 (IgG4), respectively.

‡ This number indicates the fraction of structures in the cluster with the Fc domain in the position depicted downwards. In a fully ambiguous case with no distinction between Fab and Fc, the Fc domain would be found equally in the three domain positions, corresponding to a 33% Fc occupancy.
