# Supplementary material for: In-depth analysis of subclass-specific conformational preferences of IgG antibodies
Source: IUCrJ. 2015 Jan 1;2(Pt 1):9–18. doi: 10.1107/S205225251402209X (PMC4285876; doi:10.1107/S205225251402209X)
Supplement: Supplementary file 1 [file m-02-00009-sup1.pdf]

IUCrJ

Volume 2 (2015)

Supporting information for article:

In-depth analysis of subclass-specific conformational preferences of IgG antibodies

Xinsheng Tian, Bente Vestergaard, Matthias Thorolfsson, Zhiru Yang, Hanne B. Rasmussen and Annette E. Langkilde

Supporting information

In-depth analysis of subclass-specific conformational preferences of IgG antibodies

XINSHENG TIAN[a], BENTE VESTERGAARD[a], MATTHIAS THOROLFSSON[b], ZHIRU YANG[c], HANNE B. RASMUSSEN[b], ANNETTE E. LANGKILDE[a]

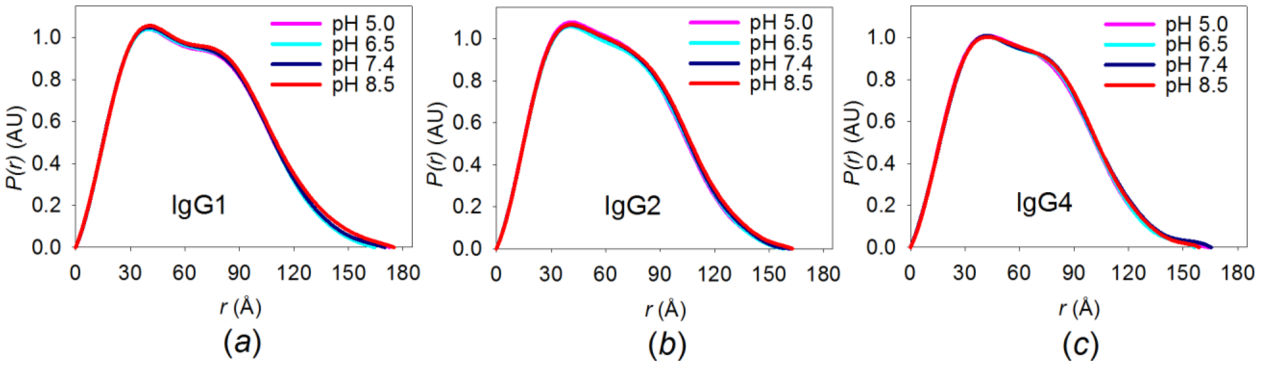

Figure S1. The pair distance distribution functions from the indirect Fourier transformation of the scattering intensity. The SAXS data were collected at pH 3.3, pH 5.0, pH 6.5, pH 7.4, and pH 8.5 as published (Tian *et al.*, 2014).

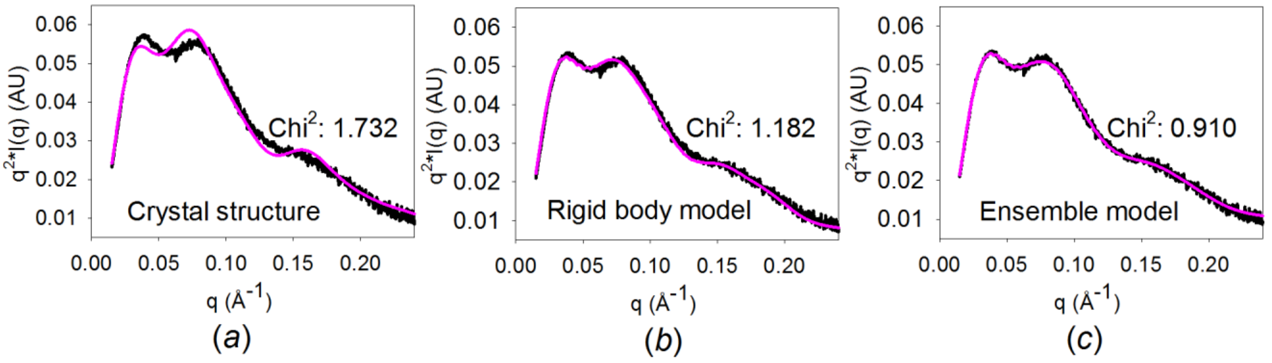

Figure S2. Comparison of the Kratky plots between experimental (blue) and theoretical (pink) SAXS curves of IgG1: (*a*) Crystal structure (PDB entry 1HZH). (*b*) Rigid body model generated using CORAL. (*c*) Ensemble model generated using EOM.

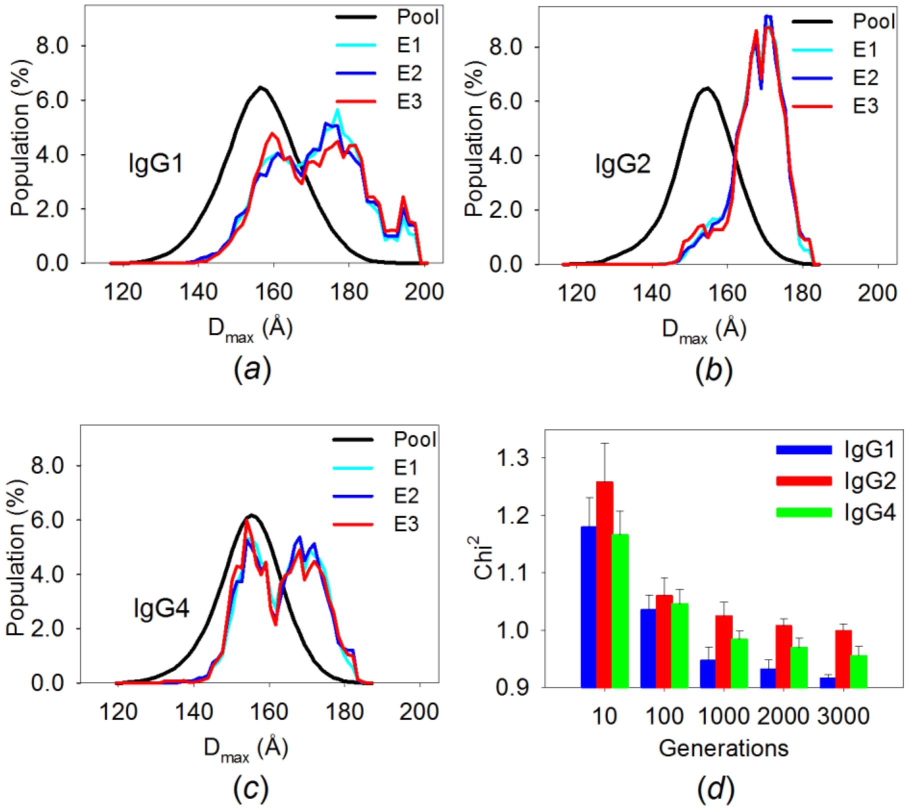

Figure S3. (*a*-*c*) Consistency of the D_{max} distribution with different GAJOE parameters. The black line indicates the D_{max} distribution of the conformers in the pool and the colored lines indicate the distribution profiles calculated by GAJOE with 1000 (E1), 2000 (E2) and 3000 (E3) generations, respectively. (*d*) The correlation between χ^2 values and generations. Each of the χ^2 value was averaged from 10 optimized ensembles with the same setting of genetic algorithm.

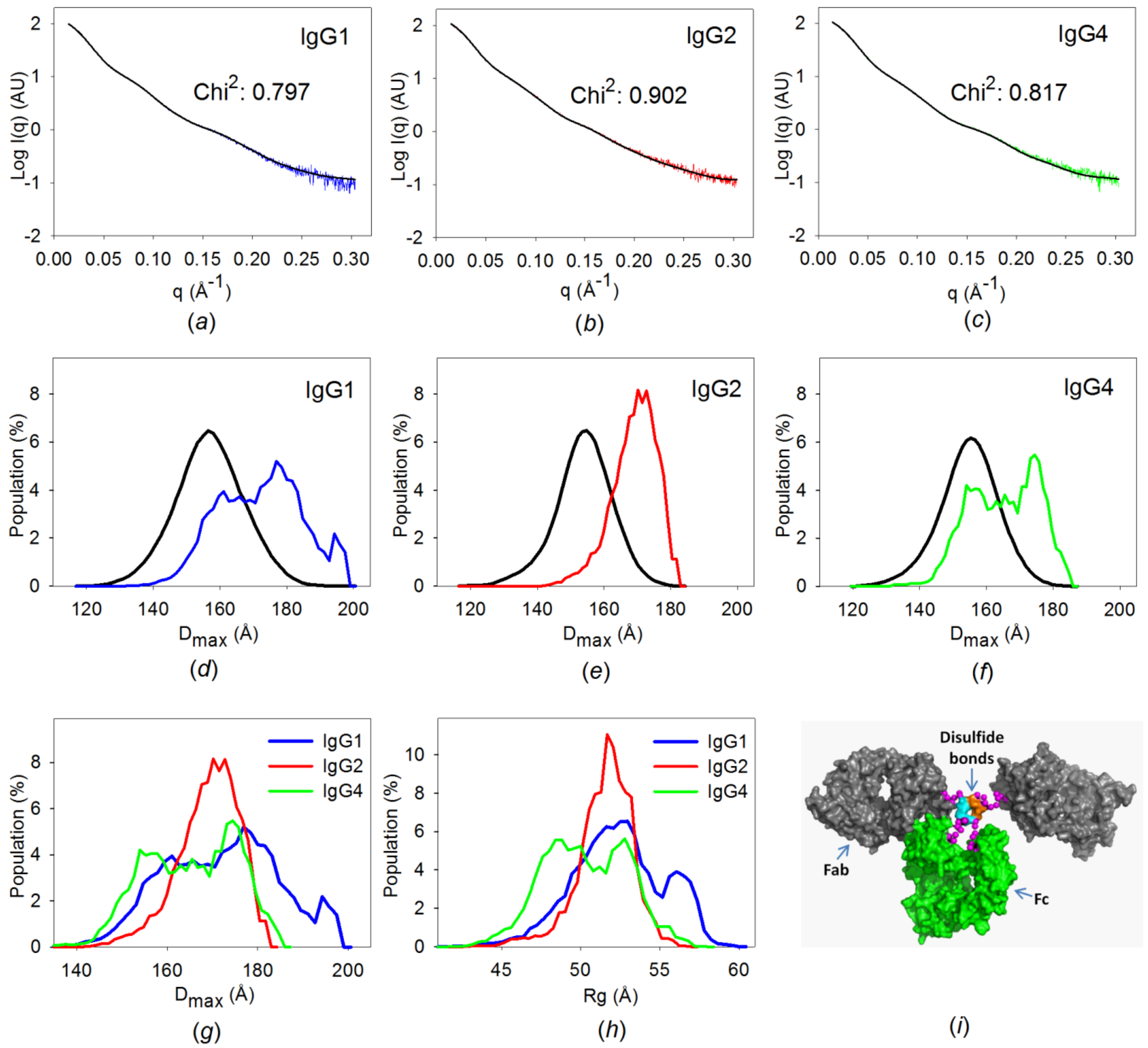

Figure S4. Results of ensemble optimization with shorter angular range (0 - 0.3 $Å^{-1}$): (*a*-*c*) The fits between the calculated scattering curve from the best ensemble (colored, selected by EOM) and the experimental data (black). (*d*-*f*) D_{max} distribution of the conformers in the pool (black) and the optimized ensembles (colored). (*g*) Comparison of the D_{max} and (*h*) R_g distributions in the optimized ensembles of three IgG subclasses. (*i*) Representative structural model of IgG1 generated by RanCh.

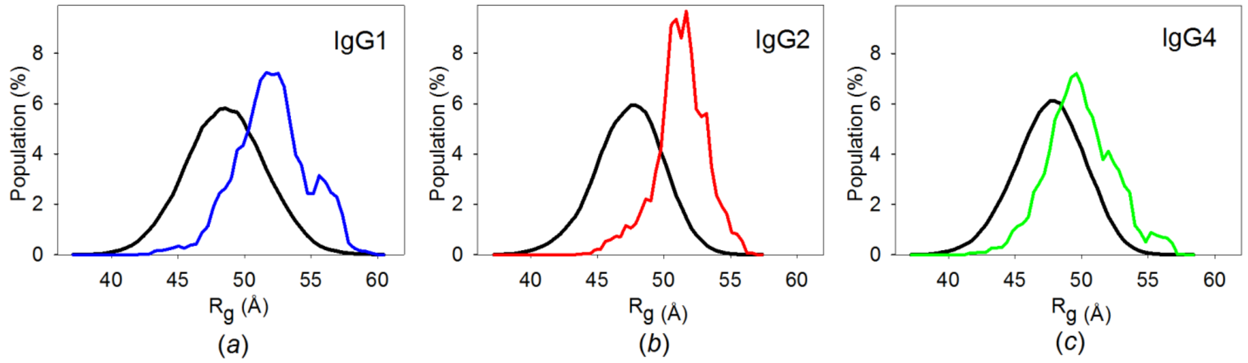

Figure S5. Additional results of ensemble optimization using an angular range of 0 - 0.5 Å^{-1}, i.e. supplementing Figure 2: *(a-c)* R_g distribution of the conformers in the pool (black) and the optimized ensembles (colored) for each IgG.

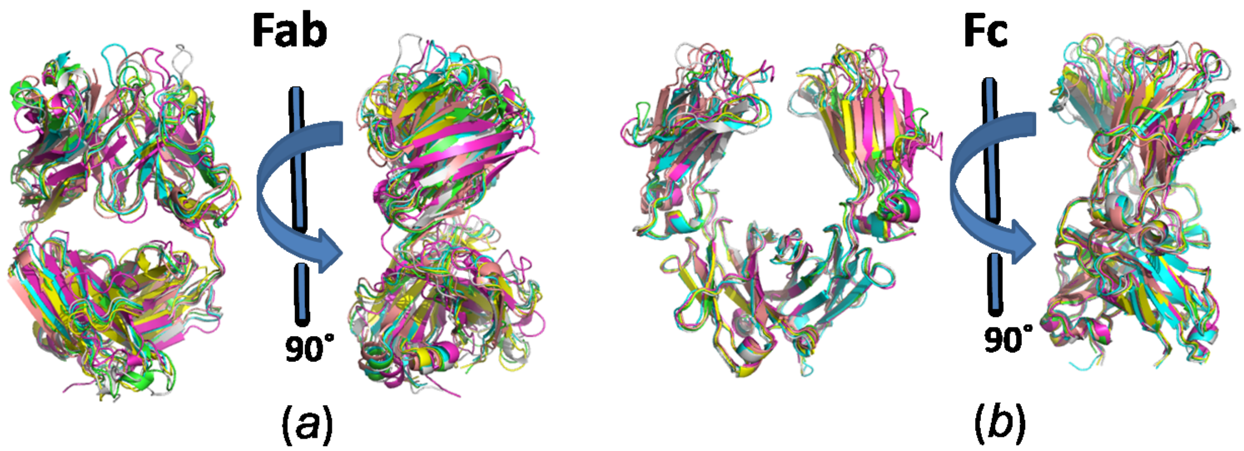

PDB	Source	Structure
4OGY	hIgG1	Complex with human plasma kallikrein
2VXV	hIgG1	Fab fragment
1AE6	mIgG2a	Fab fragment
1CLY	hIgG1	Complex withnonoate methyl ester derivative of Lewis Y (nLey)
2G75	hIgG1	Fab fragment
3W9D	hIgG1	Fab fragment

PDB	Source	Strucuture
3D6G	hIgG1	Complex with Protein-A Mimetic Peptide Dendrimer
1H3X	hIgG1	Fc fragment
1T89	hIgG1	Complex with FcrRIIIB
1HZH	hIgG1	Intact IgG molecule
4HAG	hIgG2	Fc fragment
4C54	hIgG4	Fc fragment

Figure S6. The crystal structures of Fab (*a*) and Fc fragments (*b*). Below the figures list the information of the PDB files, respectively.

Table S1. The average R_g and D_{max} of the pool and the optimized ensembles.

	IgG1		IgG2		IgG4	
	Pool	Ensemble	Pool	Ensemble	Pool	Ensemble
R_g (Å)[1]	48.6	52.0	47.4	51.3	47.6	49.9
D_{max} (Å)[2]	156.9	168.1	153.9	167.8	154.8	163.2

[1]Corresponding to the distributions shown in results in Figure S5*(a-c)*. [2]Corresponding to the distributions shown in results in Figure 2*(d-f*).

References

Tian, X., Langkilde, A. E., Thorolfsson, M., Rasmussen, H. B. & Vestergaard, B. (2014). *J. Pharm. Sci.* **103**, 1701-1710.
